# Determining the protocol requirements of in-home dog food digestibility testing

**DOI:** 10.1017/S0007114522003191

**Published:** 2023-07-14

**Authors:** Evelien Bos, Wouter Hendriks, Bonne Beerda, Guido Bosch

**Affiliations:** 1 Animal Nutrition Group, Wageningen University & Research, De Elst 1, 6708 WD, Wageningen, The Netherlands; 2 Behavioural Ecology Group, Wageningen University & Research, De Elst 1, 6708 WD, Wageningen, The Netherlands

**Keywords:** Adaptation, Faecal collection, Sample size, Protocol requirements, In-home test

## Abstract

In-home pet food testing has the benefit of yielding data which is directly applicable to the pet population. Validated and standardised in-home test protocols need to be available, and here we investigated key protocol requirements for an in-home canine food digestibility protocol. Participants were recruited via an online survey. After meeting specific inclusion criteria, sixty dogs of various breeds and ages received, during 14 consecutive days, a relatively low and high digestible complete dry extruded food containing titanium (Ti) dioxide. Both foods were given for 7 d in a cross-over design. Owners collected faeces daily allowing daily faecal Ti concentrations and digestibility of nitrogen (N), dry matter (DM), crude ash, organic matter (OM), crude fat (Cfat), starch and gross energy (GE) to be determined. Faecal Ti and digestibility values for all nutrients were not different (*P* > 0·05) from the second day onwards after first consumption for both foods. One day of faecal collection yielded reliable digestibility values with additional collection days not reducing the confidence interval around the mean. Depending on the accepted margin of error, the food and the nutrient of interest, the minimal required sample size was between 9 and 43 dogs. Variation in digestibility values could in part be explained by a dog’s neuter status (N, crude ash) and age (crude ash, Cfat) but not sex and body size. Future studies should focus on further identifying and controlling sources of variation to improve the in-home digestibility protocol and reduce the number of dogs required.

The nutritional quality of commercial dog foods is of paramount importance to the dogs’ health as nowadays these foods are the sole source of nutrients and energy for most pet dogs^(1,2)^. To evaluate the quality of (new) ingredients, formulations and processing technologies, pet food companies routinely conduct digestibility testing. It provides important information on the availability of energy and nutrients, and initial information on food acceptance as well as faecal output and consistency^(3)^. Digestibility testing by pet food companies is usually conducted at dedicated canine research facilities (kennels) where conditions are controlled, such as dog breed, housing, feeding level and schedule, and interactions with other animals and staff. Standardisation and control are important to obtain reliable, uniform and reproducible data. However, testing conditions in research facilities (kennels) differ greatly from those that dogs experience in households and as such, results may not be representative for the pet dog population for which the foods are intended. Findings will be more representative when these are obtained directly in dogs that make up the target population.

The population of pet dogs differs from kennel dogs by a greater variability in factors that impact nutrient digestibility, like age, breed, body size, neuter status, body condition and physical activity^(4–11)^. The home environment of dogs and involvement of the owner (‘in-home’^(12,13)^) may cause further variability due to owner (non)compliance, environmental conditions (e.g. feeding schedule, ambient conditions, presence of other pets in the household) and the dogs’ food history. The expected variability in conditions of in-home testing compared with those at a dedicated canine test facility will affect protocol requirements and likely necessitate adjustments of the period of adaptation to the food, faecal collection period, number of animals and more.

Digestibility testing protocols for dog food, using standardised conditions, have been developed and are widely adopted within the industry for many years^(14,15)^. Current protocols when using an indigestible marker include a minimum of six healthy fully grown dogs over 1 year of age, with FEDIAF (2020) requiring a study length of 7 d while AAFCO (2020) prescribes 10 d. The recommended study length includes an adaptation period and a faecal collection period. The AAFCO advises an adaptation period of 5 d, whereas the FEDIAF shortened this to 3 d based on a single study^(16)^, in which six beagle dogs did not show different digestibility values between pooled faecal samples over days 4–7, 8–14 and 5–21. The adaptation period aims to achieve steady-state conditions including a constant marker excretion in the faeces^(17)^, and adaption of the dogs’ digestive system to the test food^(18)^. The variation in these parameters could be strong among pet dogs, which would affect the required length of the adaptation period. For example, the body size of the dog relates directly to the transit time, defined by the average time that the marker takes to pass through the gut^(19)^. In addition to the required length of the adaptation period, it is unclear how many faecal collection days are sufficient. Hagen-Plantinga *et al.* ^(12)^ developed an in-home digestibility study using titanium (Ti) dioxide as a marker, a 7 d adaptation and 24-h faeces collection period. The authors reported that it could be questioned whether the 24-h collection period was sufficient to cover day-to-day variation in faecal marker and nutrient excretions. A minimal trial duration will improve the ease of tasks requested from the owner, thereby improving compliance to the protocol, reliability of the data and study completion. Finally, as the factors that can affect digestibility values are less controlled with in-home tests and, consequently, yield greater variability compared with tests in kennels, the former require a larger number of dogs to achieve the same precision as obtained with six dogs prescribed by the FEDIAF^(14)^ and AAFCO^(15)^ protocol.

The present study aimed to generate information on key trial variables to accurately determine in-home nutrient digestibility of canine foods. By assessing the degree of variation in food digestibility values in privately owned dogs across days, recommendations are made regarding minimal length of the adaptation period, faecal collection period and number of dogs required. We tested if the study duration with privately owned dogs could be reduced from the 2020 FEDIAF and AAFCO protocol for kennel dogs, and to what extent the number of dogs required need to be increased to provide accurate and precise dietary nutrient digestibility values.

## Experimental methods

The study design and procedures were assessed and approved by the Animal Welfare Body of Wageningen University (Wageningen, The Netherlands). The latter body judged the study to not adhere to the definition of an ‘animal experiment’ according to the Dutch Experiments on Animals Act (2014). In addition, the surveys used in this study were approved by the privacy officer of Wageningen University & Research, did not interfere significantly with normal daily life of human participants and did not include questions that were psychologically burdening. This exempts the study from review by our ethics committee, according to the guidelines of Wageningen University Medical Ethics Review Committee (Medisch Ethische Toetsingscommissie van Wageningen University, METC-WU).

### Study design

The digestibility test was conducted in-home with dog owners receiving two differently formulated, dry extruded foods and collecting their dogs’ faeces on a daily basis. A cross-over design was used with two 7 d feeding periods without an adaptation period at the start of the study or during the transition to the other diet. Each dog owner was assigned to start with one of the two foods based on the order after eligibility assessment. The study was conducted from November 2019 to August 2020 and participants started the in-home digestibility study within ±2 weeks after they were found to be eligible, resulting in different start dates per participant. An informed consent was obtained from each owner prior to the start of the study. The sample size for this study was based on feasibility, and we aimed for the maximum number of participants that could be included with the available time and means required to run the study (e.g. logistics, sample processing, chemical analyses).

### Participants

Recruitment of participants occurred through widespread advertisement of an online survey using Microsoft Forms, and the inclusion criteria were that the owner was willing to feed their dog the test foods solely and collect their dogs’ faeces. Also, dogs had to be older than 1 year, not pregnant or lactating and healthy, that is, being free of chronic disease as diagnosed by a veterinarian, medication, intestinal disorders in the previous 3 months and food allergies or intolerances.

Participants received an information brochure including the study details and were requested to weigh their dog at home or at a veterinary clinic. Participants were visited, provided with the study materials and given an oral explanation regarding the background and importance of the research, their tasks and the use of the materials. The latter included daily food portions, faeces collection bags, zip-lock bags, freezer containers, a mini freezer (Primo DV2-WS, Primo Elektro) if requested, and a diary product. The diary product included the Waltham Faeces Scoring Chart^(20)^, and owners were instructed how to score faeces for consistency. During the digestibility study, participants were in close contact with the researcher by email and/or by phone.

### Diets and feeding

The two dry extruded dog foods used (Jonker Petfood BV; Table 1) differed in ingredients and nutrient composition and were formulated to meet the nutritional guidelines of FEDIAF for adult dogs^(21)^ and to differ substantially in gross nutrient digestibility (Food A high and Food B low digestible). Both foods contained titanium (Ti) dioxide (Hombitan FG, Venator Germany GmbH) as an indigestible marker, which was devoid of particles < 100 nm based on a particle size analysis (Mastersizer 3000, Malvern Panalytical BV). Dry ingredients and the marker were mixed for 70 s using a paddle shift mixer (Forberg F60, Forberg International AS), followed by extrusion using a co-rotating double screw extruder (Baker Perkins MF50, Baker Perkins), oven-drying at 45°C overnight and coating with poultry fat (Food A and Food B) followed by a digest (Food B) using the paddle shift mixer at the research facilities of Wageningen University (Wageningen, The Netherlands).

**Table 1.** Ingredient and analysed chemical composition and energy contents of dry extruded dog Food A and Food B

Component	Food A	Food B
Ingredient composition (g/kg)		
Pre-gelatinised maize starch	434·86	–
Barley	–	327·51
Rice	43·25	299·70
Meat bone meal 61	167·42	–
Lamb-sheep meal	–	119·88
Chicken meal	123·17	102·05
Maize	83·21	–
Poultry fat	49·95	45·47
Greaves 83+	45·25	–
Beet pulp	–	34·97
Brewer’s yeast	–	19·98
Fish meal	–	19·98
Digest	–	9·99
Dog premix	13·28	9·99
Vitamin premix	13·28	–
Sepiolite	13·28	–
Egg powder	–	5·00
Lecithine	7·28	2·00
Choline chloride	4·79	1·50
Flax seed	–	1·00
TiO_2_	1·00	1·00
Chemical constituents (g/kg)		
DM	916·3	902·1
Crude ash	72·1	69·3
Organic matter	927·9	930·7
Nitrogen	42·2	34·6
Crude protein	263·4	216·5
Crude fat	108·3	91·9
Starch	380·6	401·0
Total dietary fibre	79	122
Ti	0·53	0·57
Energy MJ/kg DM		
Gross energy	18·6	17·8

TiO_2,_ titanium dioxide; Ti, titanium.

Both foods (formulated to contain A 1542 and B 1449 MJ/100 g) were fed at maintenance energy requirements^(21)^ (480 kJ × kg BW^0·75^). Feeding levels were discussed with the owner prior, or when requested during the study, and adjusted where appropriate, for example to account for a dog’s physical activity. Dog owners were provided with daily food portions, instructed to give no other foods/treats to their dog and to carefully collect and store any leftovers each day. Water was requested to be available *ad libitum* for dogs in the households.

### Faeces and data collection

Dog owners were requested to collect their dogs’ faeces twice a day during the 14-d study, preferably as clean as possible (e.g. without grass, leaves, sand) using a collection bag and with several hours between the two collections. The collection bag with faeces was placed in a zip-lock bag, which was labelled with the dogs’ name, date and time. Labelled bags were stored in containers in the freezer (–18°C) of the owners or in the freezer provided to the owner. Faeces were transported from the households within 3 weeks to Wageningen University and stored at −20°C pending further processing and chemical analyses.

Dog owners were requested to keep a diary with daily information about the dog’s consumption of the food and (accidental) other items, faeces characteristics (number of defecations, faeces consistency score^(20)^ and additional particularities) and the activity of the dog (minutes of walking and activity score on a 1–5-point scale from very inactive to very active).

### Chemical analyses

For each dog, the collected faeces were pooled per day, resulting in seven samples per period/food. Faeces were oven-dried at 60°C to a constant weight, cleared of visible contaminants if present, and ground to pass a 1-mm sieve in an ultra-centrifugal mill (ZM100, Retsch B.V.). Faecal samples were analysed using the near-infrared reflectance spectroscopy (Anadis Instruments Benelux BV & Nirvention BV), calibrated by chemical analyses of a subset of fifty faecal samples obtained in this study. Proximate analyses for the subset of faeces samples, as well as Food A and B, included in duplicate determination of dry matter (DM)^(22)^, crude ash^(23)^, nitrogen^(24)^ (N), crude fat^(25)^ (CFat), starch^(26)^ and gross energy^(27)^ (GE). Food samples were analysed for total dietary fibre^(28)^. Ti concentrations in foods and all faecal samples were determined using inductively coupled plasma-optical emission spectrometry (Iris intrepid II XSP, Thermo Fisher Scientific, Inc.) after destruction with H_2_SO_4_ using a microwave digestion system (MARS 6, CEM Corporation).

### Calculations

Organic matter (OM) content in diets and faeces was calculated as 100-crude ash. Apparent faecal digestibility (%) of nutrients was calculated^(14,15)^:

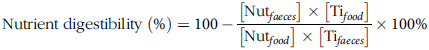

where Nut_*faeces*_, Nut_*food*,_ Ti_*faeces*_ and Ti_*food*_ are the nutrient content (% DM) and Ti content (% DM) of faeces and food, respectively. Negative digestibility values, which were predominantly observed on day 1, were omitted from the dataset (*n* 264 out of 4806). To simulate how nutrient digestibility values vary with the number of faecal collection days, the digestibility values across subsequent faecal collection days were averaged, yielding a new dataset of pooled digestibility values over 2–6 collection days.

### Statistical analyses

Data were statistically analysed using SAS (version 9.4, SAS Institute Inc.). To investigate the required duration of the adaptation and faecal collection periods, variation in faecal Ti concentrations and digestibility values was assessed by a repeated-measures ANOVA using the Proc MIXED procedure. Time effects were analysed separately for each combination of feeding period (1, 2) and food (A, B). As feeding periods 1 and 2 differed in food consumed before the period started (in-home provided food in period 1 *vs*. experimental food in period 2) and, consequently, in starting values of faecal Ti concentrations, separate analyses were considered more fitting. The effects of feeding period (1, 2) and food (A, B) were analysed separately as follows. Day was used as a REPEATED model statement^(29)^ using a first-order autoregressive covariance structure [AR(1)]^(30)^ according to 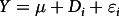 , where *Y* is the dependent variable, μ is the average intercept, *D*_*i*_ is day *i* and *ϵ*_*i*_ is the error term. Differences were considered significant at a probability < 0·05, with posthoc pairwise comparisons using the Tukey’s test. To investigate the required number of adaptation days in more detail, digestibility values of days 1, 2 and 3 were compared with a pooled sample over days 4–7, following the FEDIAF protocol, using the same model as above. A pooled sample over days 4–7 was created by averaging the digestibility values per dog over days 4–7. In addition, the effect of the number of faecal collection days in combination with the effect of different sample sizes on the precision of digestibility estimates was assessed using bootstrap sampling with 10 000 replicates.

Dog characteristics may explain variation in digestibility values, which were analysed by repeated-measures ANOVA using Proc MIXED on the complete data across feeding periods and foods, but limited to the study days after a constant marker excretion in the faeces was reached. The best model to explain the variance of the dependent variables DM, crude ash, OM, N, Cfat, starch and GE digestibility was selected using stepwise regression with the GLMSELECT procedure and the Schwarz Bayesian information criteria. The independent variables were the test food (A, B), period (1, 2), sex (female, male), neuter status (intact, neutered), body size (small, medium, large), age (young, adult, old) and their interactions. Dogs of small body size had a BW < 10 kg, medium size dogs between 10 and 25 kg and large size dogs > 25 kg. Young dogs were between 1 and 2 years and adult dogs between 3 and 7, with older dogs > 7 years of age.

## Results

### Participants and owner compliance

Sixty dogs from fifty-seven owners started the digestibility study (see online Supplementary Fig. S1 for a flow chart), of which fifty-three dogs from fifty-one owners completed the study. Dogs dropped out because the study was too time consuming for the owner (*n* 1), dogs were not eating the provided foods (*n* 4) or dogs suffered health issues unrelated to the foods (*n* 2). One dog completed half of the trial due to disliking one of the foods (A in period 1) and one dog received only one of the foods (A) due to a suspected allergy. Detailed characteristics of participants in the study are presented in online Supplementary Table S1.

As reported by the owners, other matter was consumed by forty-one dogs on 129 d out of the total of 728 study days, including dog snacks and human food products (*n* 32 dogs, on 64 d), grass and animal faeces (e.g. horse, cat) (*n* 23 dogs, on 84 d). Faecal collections were not obtained on 41 d, by fifteen owners. Out of the 687 pooled daily faecal samples, 201 contained some other matter such as leaves, grass or sand.

### Food transitions

The abrupt transition to the experimental foods at the start of the trial, and the switching between foods A and B, did not seem to cause adverse effects. One owner, participating with two dogs, reported that both dogs did not feel well on days 2 and 3 during the first period, when both dogs received Food A. This owner reported vomiting once and twice during these days. Four more dogs were reported to have vomited on days 2 (*n* 1, Food A), 4 (*n* 1, Food A), 5 (*n* 2, Food A and Food B), 6 (*n* 1, Food A) and 7 (*n* 1, Food A), of which one owner noted that the warm weather > 30°C may have been the cause. Extreme faecal consistency scores (indicated as 1 and 5) were rare (*n* 12) with values of 1·5 and 4·5 recorded 51 times out of 1439 scores.

### Variation in titanium concentrations and apparent digestibility values

**Fig. 1.**
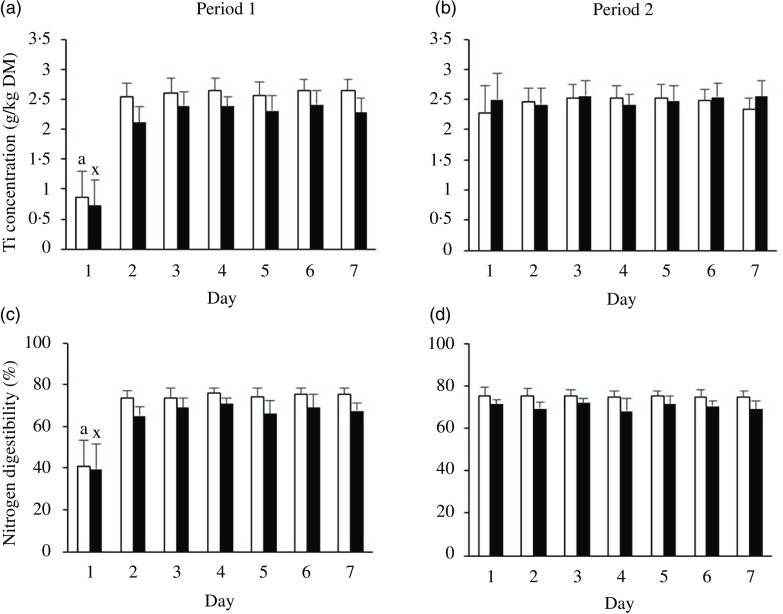
Mean daily faecal titanium (Ti) concentrations of dogs fed Foods A (□) and B (■) in (a) period 1 (A, *n* 29; B, *n* 24) and (b) period 2 (A, *n* 24; B, *n* 28) and daily nitrogen faecal apparent digestibility of the two foods in (c) period 1 and (d) period 2. Values within food (A, B) with a superscript (a, x) differ (*P* < 0·05) from corresponding values. Error bars are standard errors of the mean.

The faecal Ti concentrations collected during period 1 (29 dogs Food A, 24 dogs Food B) increased from day 1 to 2, for both foods (*P* < 0·001; Fig. 1(a)) and remained constant from day 2 onwards. Faecal Ti concentrations in period 2 (24 dogs Food A, 28 dogs Food B) were constant from the first day onwards (Fig. 1(b)). The faecal N digestibility values on day 1 in period 1, for both foods, were different from the values obtained on the other days within that period (*P* < 0·001; Fig. 1(c)). Constant faecal N digestibility values were found in period 2 from day 1 onwards for both foods (Fig. 1(d)). Similar results were found for the digestibility values of DM, OM, Cfat, starch and GE (online Supplementary Fig. S2).

In the scenario where pooling of faecal samples was simulated, digestibility values of N, DM, crude ash, OM, Cfat, starch and GE on days 2 and 3 were not different (*P* > 0·05) to the pooled samples over days 4–7 in period 1. In period 2, digestibility values at days 1, 2 and 3 were not different (*P* > 0·05) to the pooled samples over days 4–7.

Predicted LSmean digestibility values for Foods A and B and different dog characteristics, as based on data from days 2–7 during both periods, are shown in Table 2. Digestibility values varied between the two test foods for N (*P* < 0·001), DM (*P* < 0·001), OM (*P* < 0·001), Cfat (*P* < 0·001), starch (*P* = 0·047) and GE (*P* < 0·001). Male dogs tended to have higher digestibility values for OM (*P* = 0·087), Cfat (*P* = 0·086) and GE (*P* = 0·081) and neutered dogs showed higher digestibility values for N (*P* = 0·027) and lower digestibility values for crude ash (*P* = 0·039) compared with intact dogs. Age tended to increase digestibility values for DM (dogs >7 *v*. 1–2 year; *P* = 0·095) and OM (dogs > 7 *v*. 3–7 year; *P* = 0·094) and significantly increased values for crude ash (all age categories *P* < 0·05) and Cfat (dogs > 7 *v*. 3–7 year; *P* = 0·037).

**Table 2.** Factors explaining variation in the apparent faecal digestibility values (%) including days 2–7 from both feeding periods for DM, organic matter (OM), nitrogen (N), crude fat (Cfat), starch, crude ash and gross energy (GE). Factors include the test food, sex, neuter status, age and body size of the dogs. Values are least square means ± standard error

	DM		OM		N		Cfat		Starch		Crude ash		GE	
Factor	Mean	se	Mean	se	Mean	se	Mean	se	Mean	se	Mean	se	Mean	se
Food														
A (*n* 53)	78·3	0·7^a^	83·1	0·6^a^	74·2	0·7^a^	93·4	0·4^a^	98·6	0·05^a^	n.i.		81·7	0·7^a^
B (*n* 52)	74·8	0·7^b^	78·9	0·6^b^	67·9	0·7^b^	91·6	0·4^b^	98·7	0·05^b^			76·6	0·7^b^
Sex														
Female (*n* 34)	75·7	0·8	80·1	0·6	70·2	0·8	92·0	0·3	98·5	0·06	28·1	1·4	78·2	0·7
Male (*n* 19)	77·51	0·9	81·9	0·8	72·0	0·9	93·0	0·5	98·5	0·06	31·0	1·9	80·1	0·8
Neuter status														
Intact (*n* 12)	75·6	1·0	n.i.		69·6	1·1^a^	n.i.		n.i.		31·9	1·9^a^	78·3	1·0
Neutered (*n* 41)	77·5	0·6			72·5	0·6^b^					27·1	1·9^b^	80·0	0·6
Age														
1–2 year (*n* 15)	75·5	0·9	80·4	0·4	n.i.		92·3	0·5^ab^	n.i.		21·5	2·6^a^	n.i.	
3–7 year (*n* 27)	75·9	0·7	80·0	0·6			91·8	0·4^a^			29·5	1·4^b^		
> 7 year (*n* 11)	78·3	1·1	82·6	1·1			93·5	0·6^b^			37·5	2·2^c^		
Body size														
0–10 kg (*n* 10)	n.i.		n.i.		n.i.		n.i.		n.i.		21·7	2·85^a^	n.i.	
10–25 kg (*n* 23)											35·1	1·9^b^		
> 25 kg (*n* 20)											31·8	1·5^b^		

n.i., not included in the ANOVA after model selection using stepwise regression.

^a,b,c^ Values with different superscripts within a factor and nutrient differ (*P* < 0·05).

### Number of faecal collection days and number of dogs

**Fig. 2.**
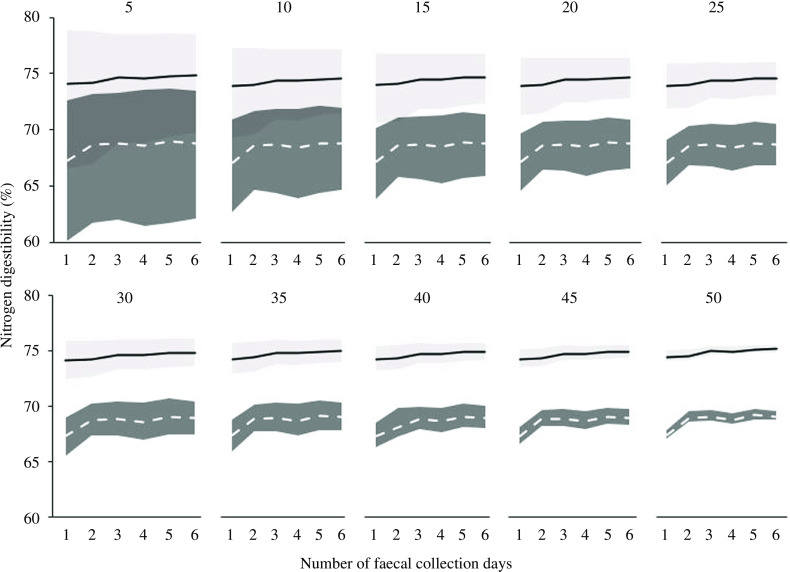
Bootstrapped estimates and confidence intervals of nitrogen digestibility for Foods A (- - - -) and B (–––) with increasing number of faecal collection days (1–6) and dogs (5–50). Bootstrap sampling included 10 000 replicates. One day represents the first accurate faecal collection day (day 2 after feeding Food A or B) with 2–6 days representing calculated values from the addition of subsequent collection days (days 3–7).

The use of faecal samples of day 2 only, as opposed to those of multiple days, did not significantly increase variation in the digestibility estimates. Bootstrap analyses compared data from only day 2 to those for multiple days, as created by pooling (i.e. days 2–3 up to days 2–7), and this produced consistent confidence interval widths (Fig. 2 for N digestibility; online Supplementary Fig. S3 for the other nutrients). In the scenarios of increasing the number of dogs, the bootstrap analyses showed decreasing variation and reductions in confidence interval widths, both for Foods A and B (Fig. 2; online Supplementary Fig. S3).

## Discussion

In-home dog food digestibility testing requires validated protocols that are tailored for settings with privately owned dogs in a home environment. We studied variation in dog food digestibility values obtained in-home and provide information on the minimal number of adaptation and faecal collection days to obtain reliable apparent faecal digestibility estimates of dietary nutrients and energy. The study population, in terms of sample size and dog characteristics, was assessed for determining the variability of the digestibility estimates, which sheds light on the minimal number of animals required to test specific digestibility differences between foods or to test a food with *a priori* set acceptable margin of error.

### Adaptation period

Food digestibility studies that use indigestible markers, like Ti, require a sufficiently long adaptation to the food to ensure accurate digestibility estimates. A constant rate of excretion of the marker in the faeces is essential, as well as adaptation of digestive processes (gut motility, enzyme secretions, absorption, microbial fermentation, etc.). The time required to reach stable faecal marker concentrations is determined by the gastrointestinal transit time of a food. The transit time of food is affected by the body size of the dog^(19)^, with 24 h reported in Dachshunds (6·3 kg BW) to 43 h in Great Danes (53·6 kg BW). Food composition also affects transit time in the intestinal tract. In English Pointer dogs (*n* 30, average BW = 17·1 kg), mean retention time of a high-fibre food was only 13 h while for a low-fibre food this was 23·4 h^(31)^. Others have reported values ranging from 20·4 to 32·7 h depending on the fibre source^(32,33)^. In Beagle dogs (*n* 6, BW range 9–14·1 kg), retention time ranged from 19·5 to 22·0 h for a fine and course diet, respectively^(34)^.

In the present study, one day of adaptation was sufficient to reach stable faecal Ti concentrations for all dogs during period 1, regardless of the digestibility of the food (Fig. 1). There was no significant correlation between BW and adaptation period. Analyses of the faecal Ti concentration data for small, medium and large dogs (Food A, *n* 6, 9, 13; Food B, *n* 4, 13, 7, respectively) showed high Ti concentrations at the second day for both foods for all body size groups. However, small dogs that consumed Food B in period 1 showed relatively high Ti concentrations on the third day (data not shown). Two of these four small dogs that were fed Food B in period 1 did not consume the test food in the morning, but in the evening, which means that variation in eating patterns between dogs, especially at the start of the study, could have delayed the increase in Ti concentrations in the faeces. Considering the importance of a stable faecal Ti excretion for accurate digestibility estimates, in-home test protocols should clearly instruct owners to feed the test food in the morning of day 1 onwards and include sufficient time to cover potential individual variation due to differences in consumption patterns, gastrointestinal transit times and defecation patterns. From the present study, a 1-d adaptation period appeared to be sufficient for Ti to reach a steady state.

The adaptation period should also ensure that the dogs’ digestive system adapts to the test food and achieves a steady state. Although digestive enzyme activity and microbiota composition in dogs adapt within a few hours after changes in the amount of dietary protein, carbohydrate or lipid^(18,35,36)^, stabilisation might take longer. The timing of such changes regarding its impact on nutrient digestibility in dogs is largely unknown, but a rapid transition from one to another food can cause gastrointestinal distress^(37,38)^. Except for one owner, participating dog owners in the present study did not report adverse effects following abrupt transition to the experimental foods at the start of the study and during the transition to the food fed in period 2. Nevertheless, gradual switching could be included in future in-home testing to prevent potential digestive upset in dogs that are more sensitive to dietary changes or to cater to the wishes of participating dog owners, resulting in an extended adaptation period.

Adaptation of the gastrointestinal tract of the dogs in the present study was not associated with changes in apparent faecal digestibility of nutrients as from the second day onwards, digestibility values of N, DM, OM, Cfat, starch and GE for both foods were found to be stable. The contrasts in nutrient composition and ingredients between Foods A and B were small. More profound digestive adaptions can be expected when transitioning to foods with a greater contrast in ingredients (e.g. those used in dry *v*. moist food), nutrient compositions and digestibility. Out of the fifty-three dogs, forty-seven dogs switched from a dry extruded food to one of the two test foods (online Supplementary Table S1). Six dogs transitioned from a meat-based to the dry extruded foods, thus experiencing a greater change in food composition. Also for these dogs, stable digestibility values from the second day onwards were observed in the present study. The dogs’ digestive system appears to adapt rapidly to novel foods, and an adaptation period of 3 d as indicated by Nott *et al.* ^(16)^ can be even shortened to 1 d to yield stable digestibility values.

### Faecal collection

Food digestibility values are often derived from faecal samples of which the composition may vary from day-to-day due to fluctuations in the metabolic activity of the gut microbiota^(39)^. Only minor metabolic fluctuations are expected when the dog has a regular eating pattern, is adapted to the test food and no other foods or treats are consumed. Non-compliance including consumption of other material (e.g. treats, food, wood, grass) or contamination of faeces samples increases day-to-day variation. Digestibility values in the present study were stable from days 2 to 7, and one day of faecal collection seems to suffice for a precise nutrient digestibility determination. Moreover, nutrient digestibility values of day 2 were not different to those determined in accordance to the FEDIAF guidelines^(14)^ from days 4 to 7. Pooling faeces from multiple collection days (at least 2 d) could guarantee greater precision than a single grab sample^(40)^. Nevertheless, in the current study the simulated pooling of samples, by using results from days 2 to 6, did not decrease confidence interval width. Additional faecal collection days, therefore, do not increase precision substantially.

It can be speculated that pooling faecal samples within a day, as practiced in the current study, covered already substantial variation within each individual dog. One faecal collection day may suffice, but it might be practical to include multiple faecal collection days in a test protocol to account for infrequent defecations, challenges to collect faeces (e.g. location, contaminations with debris) or small faecal volumes in the case of small dogs.

### Study population

The appropriate number of dogs for in-home digestibility studies relates strongly to the accepted and actual variation in digestibility values depending on whether the data are used for assessment or statistical comparisons between diets. Previously conducted apparent faecal digestibility tests of dry foods with kennel dogs in the USA (177 foods from Hall *et al.* ^(41)^ with *n* 6 dogs/food; personal communication with the authors), following the standard AAFCO quantitative collection protocols, had an average within-test margin of error (equal to half of the 95 % CI) of 1·8 % for DM, 2·2 % for CP and 1·5 % for GE. Similar average margins of error were found for similar tests in Brazil (22 studies, 6 dogs/food; personal communication Dr. A. Carciofi) with values of 2 % for DM (range 0·4–3·7 %), 1·8 % for CP (0·9–3·0 %) and 1·6 % for GE (0·5–3·1 %). These ranges in the studies by Carciofi indicate that the margin of error currently accepted for digestibility testing varies per test and nutrient of interest. Based on the results for variability of our study and implementing the averages (Hall *et al.*, 2013) and maximal (Carciofi) margins of error, the required number of dogs for in-home digestibility testing of Food A would be 9–21 for DM, 15–25 for CP and 9–24 for GE. For Food B, these numbers would be 17–35 dogs for DM, 13–23 for CP and 25–43 for GE (online Supplementary Fig. S4). These two test foods were formulated to differ in composition and digestibility, but the differences do not cover the full range in available commercial dog foods. For example, protein contents of Food A and Food B were 263 and 217 g/kg DM, and protein digestibility values were 74 and 68 %, respectively, whereas protein content can range from 164 to 440 g/kg DM and protein digestibility from 70 to 92 %^(42–44)^. To further understand the variability in in-home digestibility testing and number of dogs required in future studies, it is of interest to test more foods with different composition and digestibility values.

In addition to the test food used, the precision of the digestibility values is affected by variation that originates from study conditions (e.g. owner compliance) and test subjects. The present variation in digestibility values could be partly explained by individual dog characteristics^(4–7,9)^, including sex, neuter status and age, but not body size. Male dogs tended to have higher digestibility values for OM, Cfat and GE, which might need further validation as this is contradictory to an earlier finding by Hagen-Plantinga *et al.* ^(12)^ who reported no differences in GE digestibility between male and female dogs (overall *n* 39). Compared with intact dogs in the present study, neutered dogs showed higher digestibility values for N and lower values for crude ash, with no differences observed for DM, OM, Cfat, starch and GE. Differences between male and female dogs in nutrient digestibility may be expected, as well as between neutered and intact dogs, due to differences in physical activity, metabolism, hormones, microbiota and food intake, which all potentially affect the digestive processes^(10,45–47)^. Digestive enzyme activity has been reported similar for sexes, but sex effects exist for lipid metabolism^(48)^. To the authors’ knowledge, few data are available in the literature with regard to the effect of sex and neuter status on apparent faecal nutrient digestibility in dogs, besides the study by Hagen-Plantinga *et al.* ^(12)^ reporting an effect on energy digestibility.

Older dogs had higher digestibility values for crude ash compared with the other two age groups (old > adult > young) and higher values for Cfat compared with adults but not younger dogs, with similar trends for DM (old > young) and OM (old > adult). Other studies using dogs (*n* 24–39) in the same age range (1–13 year) as the present study did not find an effect of age on digestibility efficiencies^(12,49–52)^, and one study even found lower digestibility values for DM, Cfat and N for older dogs (10·2 (se 1·0) year; *n* 18) compared with adult dogs (2·6 (se 0·9) year; *n* 18).

### Application for future in-home testing

The use of an indigestible marker is a practical method for digestibility measurements in a relatively un-controlled environment^(12)^. Titanium dioxide (TiO_2_) has been approved as a colouring agent and feed additive^(15)^, validated as a digestibility marker for multiple animal species^(12,53–59)^ and used extensively in the past. However, recently the safety of TiO_2_ as a food additive has been questioned^(60,61)^, given the body’s potential to absorb nanoparticles. In the present study, the ingested TiO_2_ concentrations were 3·1 and 3·3 % (Food A and B, respectively) of the no-adverse effect level of 1 g/kg body weight and the TiO_2_ product did not contain nanoparticles^(60)^ (particle size > 100 nm). To provide maximum confidence to owners regarding safety, future research should investigate alternative markers to be used for in-home digestibility studies.

Dog owners can provide additional information with an in-home digestibility study including information on food appearance, pet behaviour and activity, and general satisfaction. Also, information on faecal output and food acceptance can be obtained. In the case of the latter, training programmes and practice might be required to improve the owners’ evaluation, as a recent study found inconsistencies in faecal scores using the Waltham scoring system by different individuals with varying experience^(62)^. In addition, owner compliance is critically important as non-compliance will impact on the variation in digestibility values due to, for example, the (accidental) provision of additional food to the dogs or due to faecal sample contamination (e.g. with grass, sand). The impact of non-compliance on digestibility values requires further study. Nybroe *et al.* ^(63)^ conducted an in-home digestibility study with dogs, and these authors decreased the 4-d faecal collection period as recommended by FEDIAF to 2 d in order to reduce the chance of non-compliance. The investigation of the minimal study length as done in the present study is of high relevance as owner compliance is influenced by the duration and complexity of the requested tasks^(64)^.

The digestibility values obtained in the present study could have been compared with values obtained using a digestibility study with kennelled dogs in a laboratory setting, as suggested by Plantinga *et al.* ^(12)^. In case such comparison would be made, one would then attribute potential significant differences in digestibility values between the two approaches to differences in factors such as the dog population (e.g. breed, age, sex, activity), feeding regimen, contaminations, housing conditions and more. The conclusion of such comparison would have been that the values obtained are specific for the specific situation (kennel *v*. in-home). Rather than comparing methods, further studies should, therefore, focus on identifying and controlling sources of variation for in-home digestibility testing which will lead to improved repeatability, accuracy and precision.

### Conclusion

This study provides insight in key trial variables to accurately determine in-home nutrient digestibility of canine foods and sheds new light on the digestibility test protocols currently used in canine facilities around the globe. Findings indicate the sufficiency of one adaptation and one faecal collection day for an accurate digestibility estimate. In addition, based on margin of error currently accepted for digestibility testing the required sample size for an in-home food test of digestibility would range from 9 to 43 dogs depending on properties of food and nutrient of interest.

## Supplementary material

For supplementary material accompanying this paper visit https://doi.org/10.1017/S0007114522003191. click here to view supplementary material
