# Supplementary material for: Determining the protocol requirements of in-home dog food digestibility testing
Source: Br J Nutr. 2022 Nov 28;130(1):164–73. doi: 10.1017/S0007114522003191 (PMC10244015; doi:10.1017/S0007114522003191)
Supplement: Supplementary file 1 [file S0007114522003191sup001.docx]

**Supplementary Materials**

n=146

Applied for participation

Contact established

Finished the trial

Started the trial

Selected for participation

n=74

n=53

Owner withdraws

Dog is not eligible

Dog has non-food related health issues

Dog does not eat one of the foods

Dog does not eat both foods

Owner withdraws

n=60

No response

n=91

Dog is not eligible

Owner is not eligible

**Figure 1.** Flowchart of participation of dogs (n) from owners applying for participation to number of dogs finishing the trial and reasons for exclusion in the various stages of the recruitment and trial.

**Table 1.** Characteristics of participants that finished the trial

| **Category** | **Level** | **n** |
| --- | --- | --- |
| *Dogs* |  | *53* |
| Age | Young (1-2 year)  Adult (3-7 year)  Old (>7 year) | 15  27  11 |
| Body size | Small (<10 kg)  Medium (11-25 kg)  Large (>25 kg) | 10  23  20 |
| Sex | Males  Females | 19  34 |
| Neuter status | Intact  Neutered | 12  41 |
| Body condition score^1^ | 1  2  3  4  5 | 2  6  40  4  1 |
| Breed | Pure breed  Mixed breed | 31  22 |
| Prior food type | Kibbles  Meat | 47  6 |
| Food history (number of prior foods) | 1-2  3-5  >5  Unknown | 22  20  10  1 |
| *Dog owners* |  | *52* |
| Age (years) | <20  20-29  30-39  40-49  50-59  60-69  ≥70 | 3  11  8  10  11  7  2 |
| Gender | Males  Females | 44  8 |
| Education | Primary education  Secondary education  Primary vocational education  Secondary vocational education  Higher vocational education  Scientific education | 1  9  3  15  11  13 |
| Dog housing | Always indoor  Mainly indoor  Mainly outdoor  Always outdoor | 33  16  3  0 |
| Presence of other dogs | Yes  No | 22  30 |
| Presence of other pets | Yes  No | 28  24 |
| Presence of children | Yes  No | 38  14 |
| Number of adults present | 1  2  3  4 | 11  28  9  4 |

^1^Derived from Laflamme (1997) and FEDIAF (2019)

**(A)**

**(B)**

Period 2

Period 1

a

x

**(C)**

**(D)**

ab

b

ab

b

a

ab

ab

**(E)**

**(F)**

a

x

**(G)**

**(H)**

a

x

**(I)**

**(J)**

a

x

**(K)**

**(L)**

a

x

**Figure 2.** Mean daily faecal apparent digestibility values of Food A (□) and B (■) in period 1 (A, n=29; B, n=24) and 2 (A, n=24; B, n=28) for **(A, B)** dry matter, **(C, D)** crude ash, **(E, F)** organic matter, **(G, H)** crude fat, **(I, J)** starch, and **(K, L)** gross energy. Values within food (A, B) with a superscript (a, x) differ (P<0.05) from corresponding values. Error bars are standard errors of the mean.

**(A)**

5

10

15

20

25

30

35

40

45

50

Dry matter digestibility (%)

Number of faecal collection days

**(B)**

5

10

15

20

25

30

35

40

45

50

Crude ash digestibility (%)

Number of faecal collection days

**(C)**

5

10

15

20

25

30

35

40

45

50

Organic matter digestibility (%)

Number of faecal collection days

**(D)**

5

10

15

20

25

30

35

40

45

50

Crude fat digestibility (%)

Number of faecal collection days

5

10

15

20

25

30

35

40

45

50

**(E)**

Starch digestibility (%)

Number of faecal collection days

**(F)**

5

10

15

20

25

30

35

40

45

50

Gross energy digestibility (%)

Number of faecal collection days


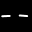

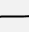
**Figure 3.** Bootstrapped estimates and confidence intervals of **(A)** dry matter, **(B)** crude ash, **(C)** organic matter, **(D)** crude fat, **(E)** starch, and **(F)** gross energy digestibility for Food A ( ) and B ( ) with increasing number of faecal collection days (1 to 6) and dogs (5 to 50). Bootstrap sampling included 10,000 replicates. One day represents the first accurate faecal collection day (day 2 after feeding Food A or B) with 2 to 6 days representing calculated values from the addition of subsequent collection days (days 3 to 7).

Number of dogs

Margin of error

Dry matter

Crude protein

Gross energy


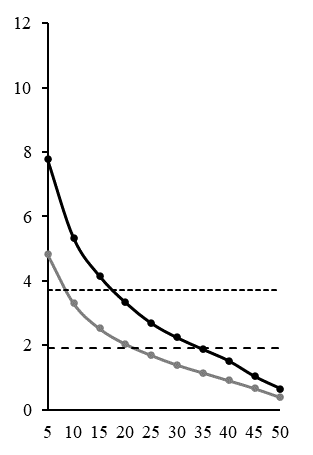


**Figure 4.** The margin of error of the bootstrapped estimates of dry matter, crude protein and gross energy for Food A and B, when increasing the number of dogs. The margin of error equals half of the 95% confidence interval. The average and maximal margin of error indicate margins found in kennel tests, adapted from Hall et al. (2013) and data from Dr. Carciofi (personal communication with the authors).
